# Supplementary material for: Naturally acquired antibody kinetics against Plasmodium vivax antigens in people from a low malaria transmission region in western Thailand
Source: BMC Med. 2022 Mar 9;20:89. doi: 10.1186/s12916-022-02281-9 (PMC8904165; doi:10.1186/s12916-022-02281-9)
Supplement: Supplementary file 1 — Additional File 1: Fig S1. Schematic representation of the relationship between the population-level and individual-level parameters for the half-life of the short-lived ASCs. Fig S2. Total IgG kinetic profiles against 52 P. vivax antigens in 34 Thai patients with symptomatic P. vivax infections. Fig S3. Adjusted total IgG kinetic profiles at an individual level following symptomatic infections. Fig S4. Comparison of the adjusted peak IgG subclass magnitude between symptomatic Thai patients and the PNG hyperimmune pool. Fig S5. Comparison of the peak IgM magnitude between symptomatic Thai patients and malaria-free individuals. Fig S6. Unadjusted IgM kinetics against 15 P. vivax antigens in 34 Thai individuals following symptomatic P. vivax infections. Fig S7. Distribution of data for estimated half-lives of antibodies and ASCs against IgG-immunogenic antigens following symptomatic P. vivax infections in the Thai symptomatic cohort. Fig S8. Comparison of adjusted total IgG against 27 P. vivax antigens following symptomatic or asymptomatic P. vivax infections. [file 12916_2022_2281_MOESM1_ESM.docx]

Additional File 1: Figures S1-S8


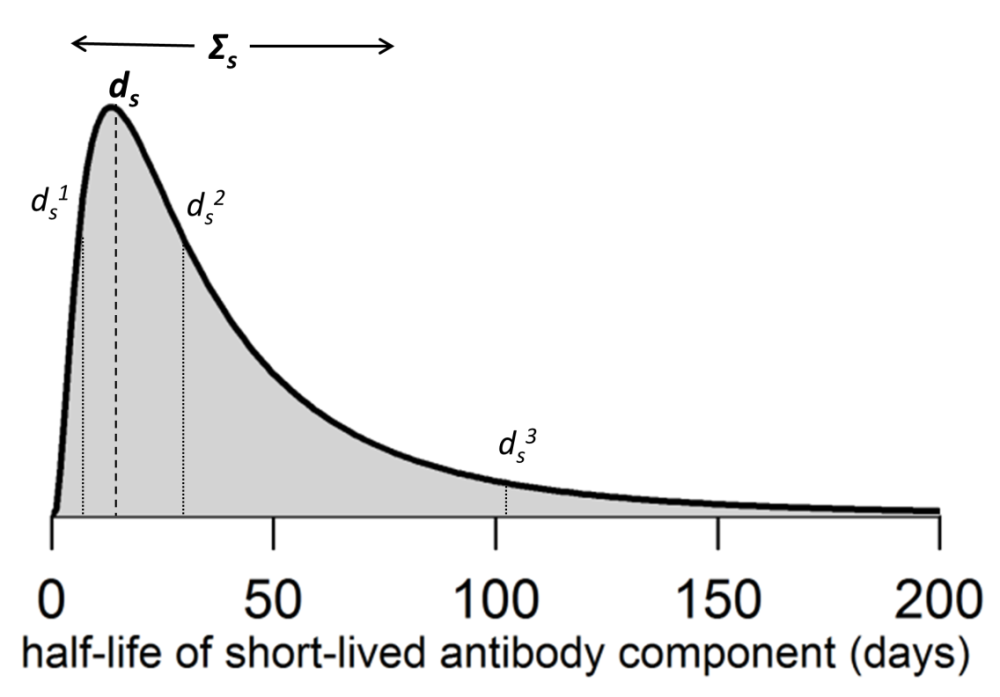


**Figure S1.** Schematic representation of the relationship between the population-level and individual-level parameters for the half-life of the short-lived ASCs. It is assumed that half-lives are log-Normally distributed throughout the population. The population-level parameters define the mean *d_s_* and standard deviation Σ_s_ of this distribution. The individual-level parameters *d_s_^n^* for each of the *n* participants follow the log-Normal distribution defined by the population-level parameters. Three representative individual-level parameters are shown for illustration.


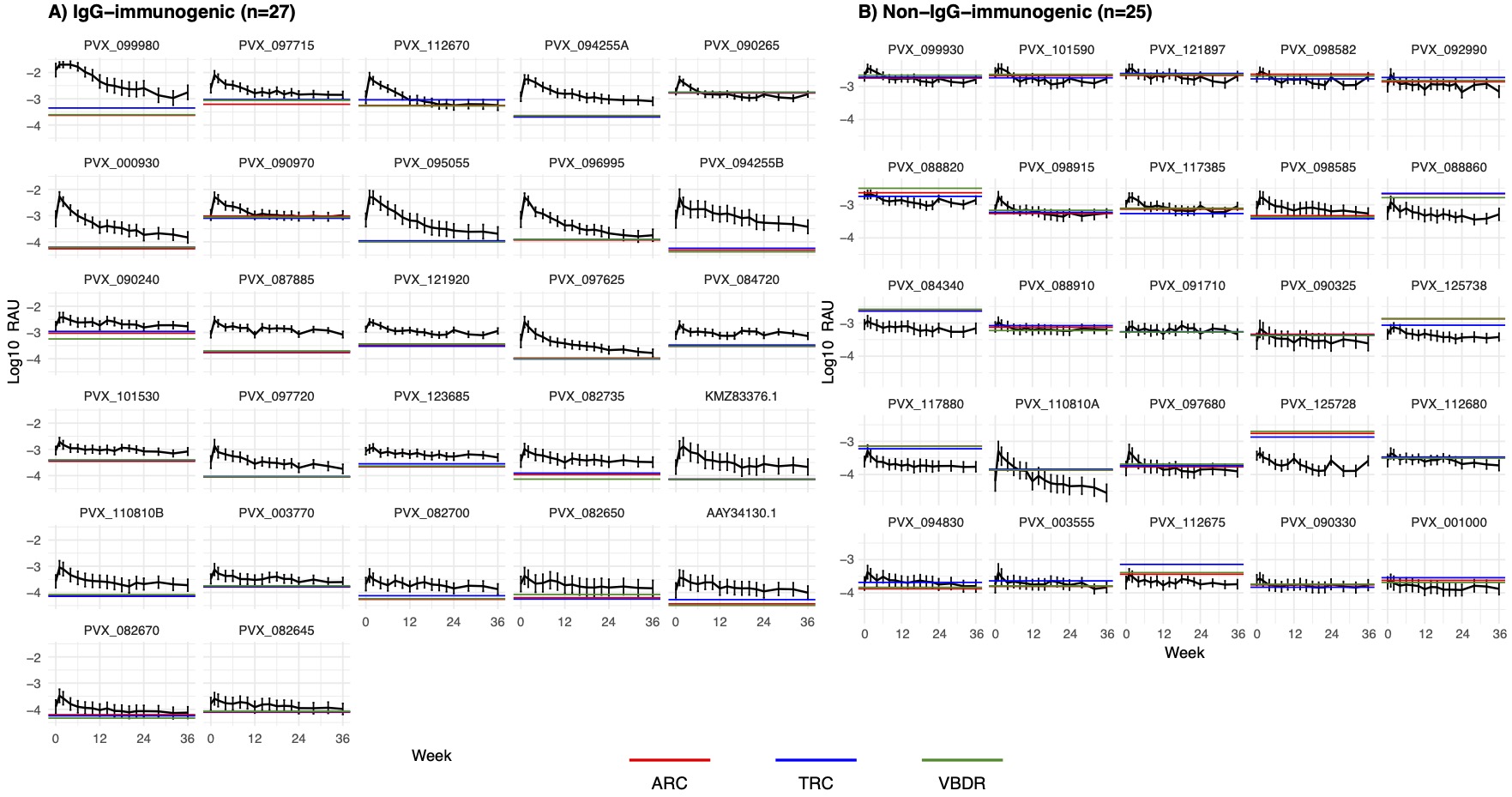


**Figure S2.** Total IgG kinetic profiles against 52 *P. vivax* antigens in 34 Thai patients with symptomatic *P. vivax* infections. Antigen-specific IgG antibody responses were measured for 9 months following a symptomatic *P. vivax* infection among 34 patients from western Thailand. The observed kinetic profiles were categorised into **A)** IgG-immunogenic (> 1 SD at 1-week post-infection above negative control baseline; n = 27) and **B)** non-IgG-immunogenic (> 1 SD at 1-week post-infection; n = 25). The horizontal lines represent the median of the negative control panels which consist of malaria-free individuals from 3 sources: Australian Red Cross (ARC; n = 100), Thai Red Cross (TRC; n = 72) and Volunteer Blood Donor Registry (VBDR; n = 102). The negative control panels are used as a background in comparison to the antibody responses observed in the symptomatic cohort. Data are expressed as median ± 95% CI of the 34 patients.


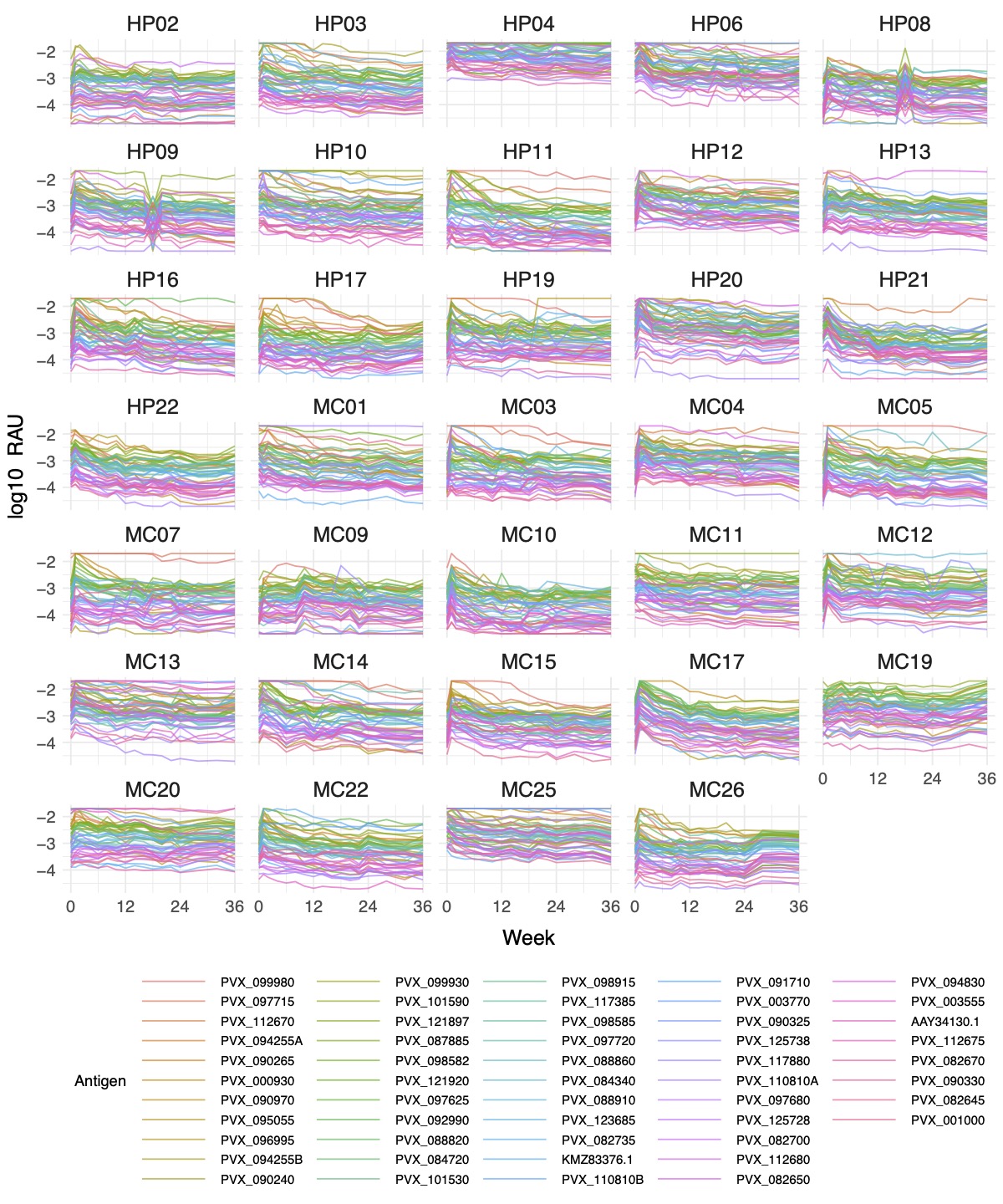


**Figure S3.** Adjusted total IgG kinetic profiles at an individual level following symptomatic infections. Antigen-specific IgG antibody responses were measured for 9 months following a symptomatic *P. vivax* infection among 34 patients from western Thailand. Each line and colour represent one *P. vivax* antigen and its specific antibody kinetics following exposure as observed in each of the 34 participants. Note that a limitation in this data is the peak for HP08 and drop for HP09 at week 18; we assume that a labelling error occurred in the field and that these two samples are swapped, however we have not altered the data to account for this.


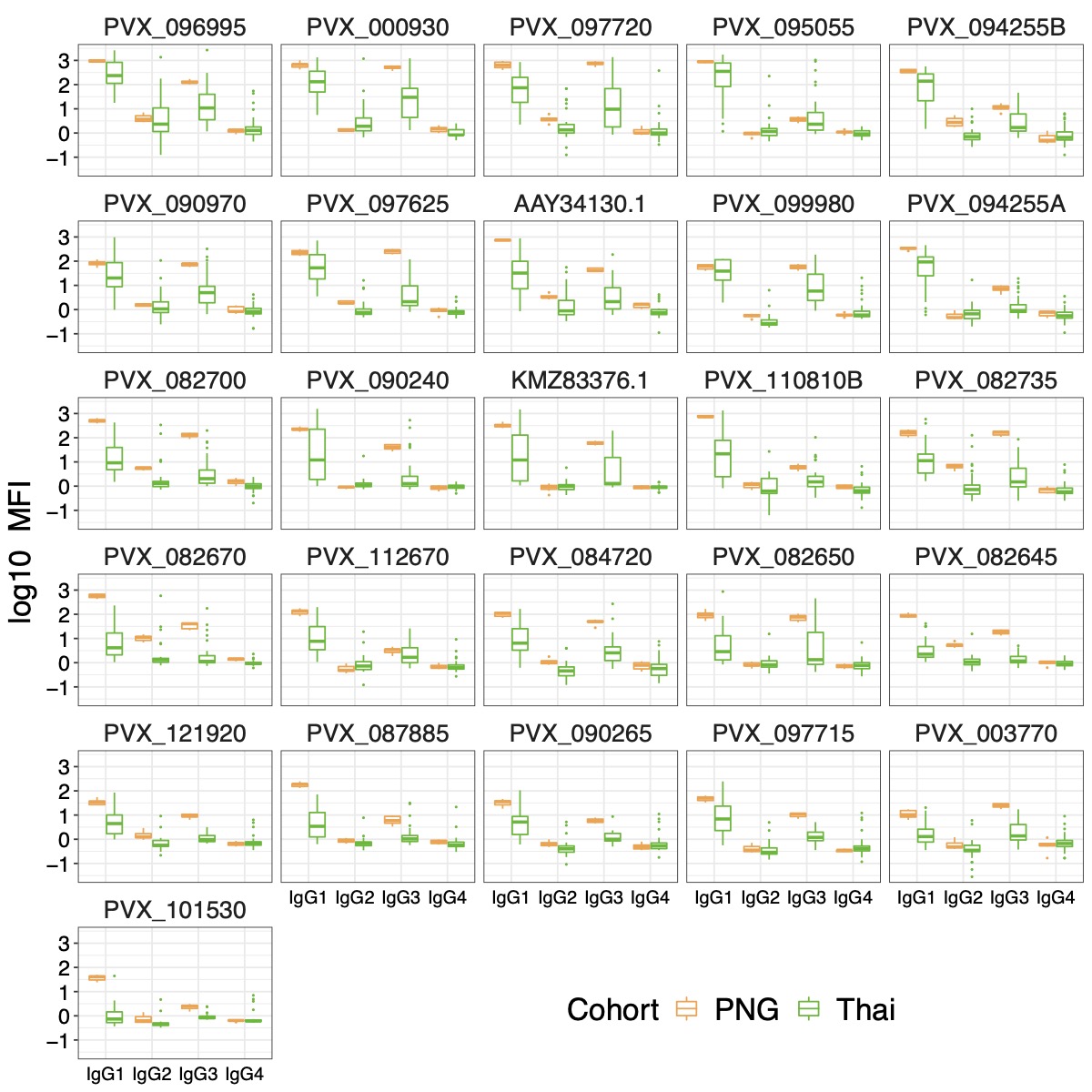


**Figure S4.** Comparison of the adjusted peak IgG subclass magnitude between symptomatic Thai patients and the PNG hyperimmune pool. Antigen-specific IgG subclass responses against 26 IgG-immunogenic (PVX_123685 was excluded) antigens were measured for 9 months, and the peak of responses (2-week post-infection) was compared between symptomatic Thai patients and the positive control (hyper-immune pooled plasma of infected PNG individuals). PNG results were collected from the standard curves included in each independent experiment of IgG subclass measurement (n=7). Data were adjusted by subtracting the median of pooled negative control panels (n=274).


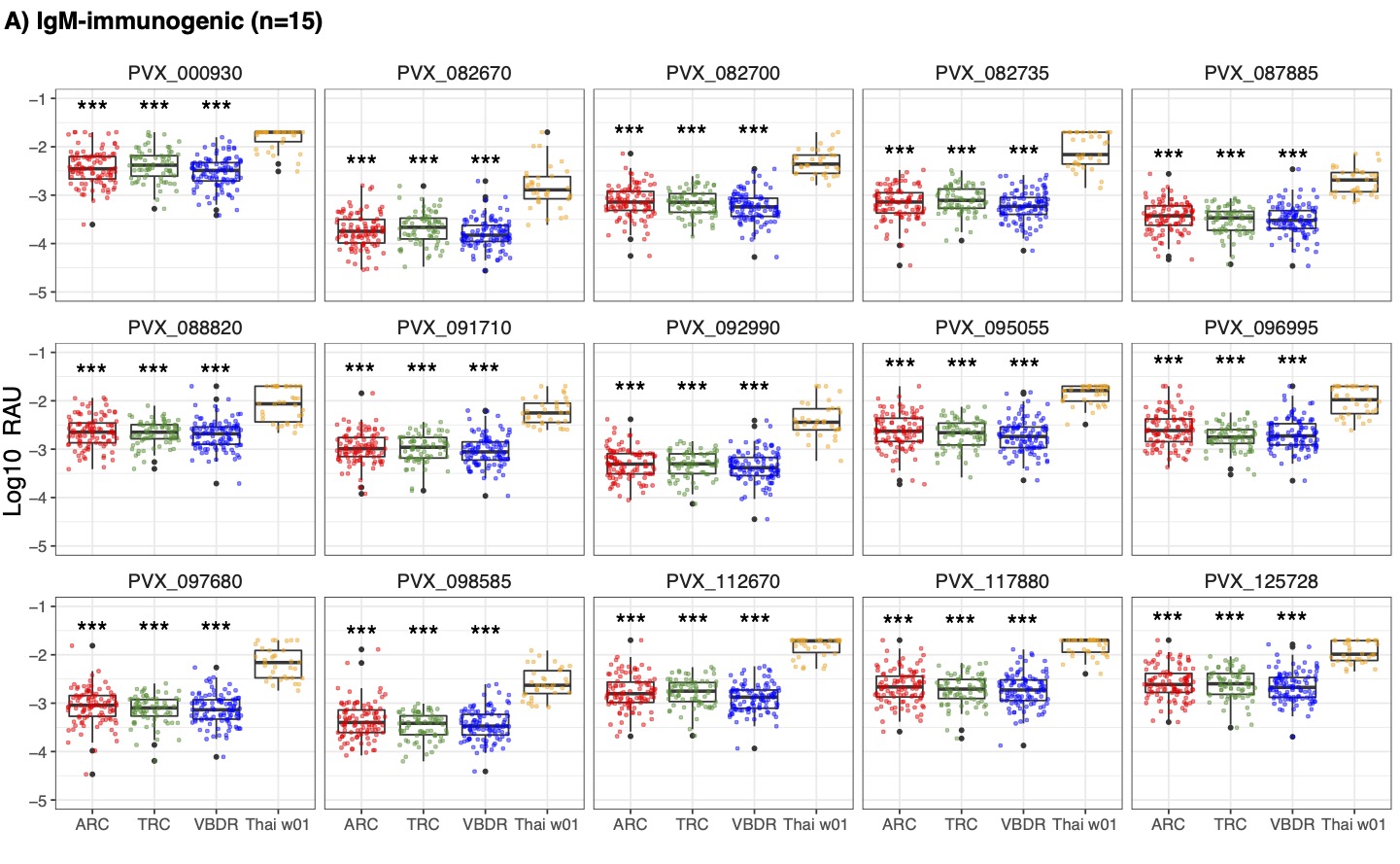


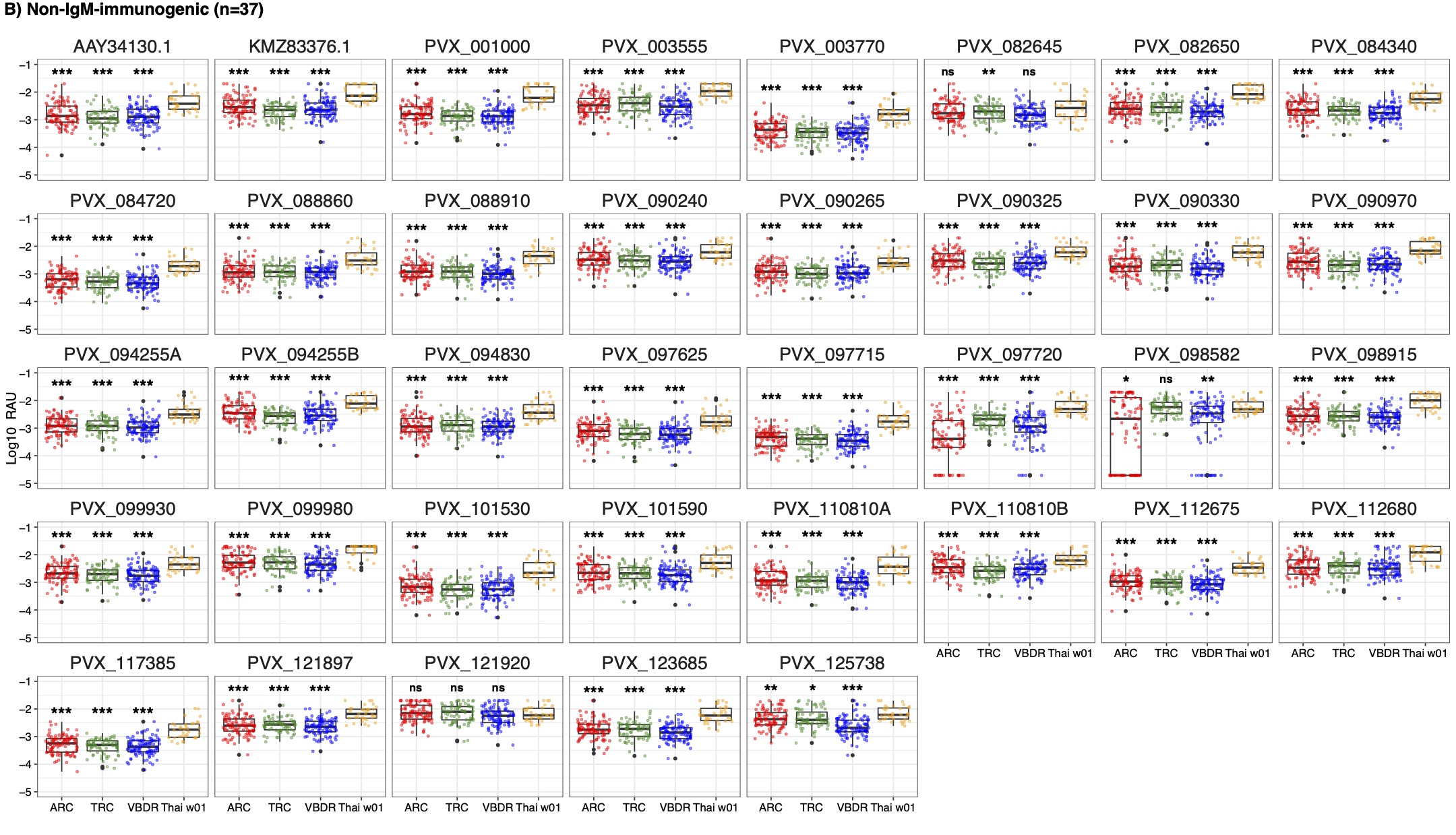


**Figure S5.** Comparison of the peak IgM magnitude between symptomatic Thai patients and malaria-free individuals. IgM level was measured at the peak of responses (1 week following a symptomatic *P. vivax* infection) among 34 Thai patients and was compared to ARC (n = 100), TRC (n = 72) and VBDR (n = 102). Mann-Whitney’s (non-parametric) test was performed (p > 0.05 ns, < 0.05 *, < 0.01 **, < 0.001 ***). To down-select IgM-positive antigens, medians and standard deviations (SD) were calculated and plotted to identify **A)** antigens with medians more than 2 SD above the pooled negative control panels (n=15) and **B)** those without (n=37).


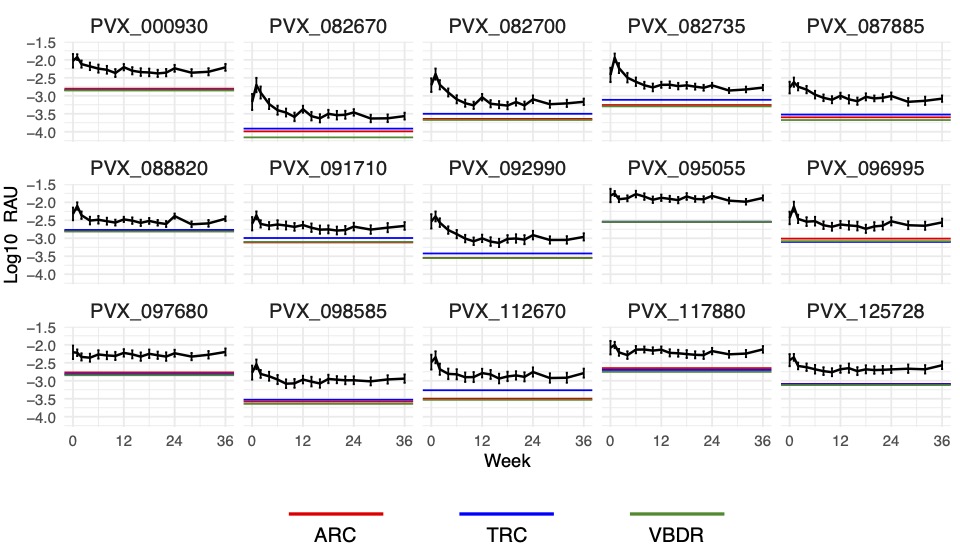


**Figure S6.** Unadjusted IgM kinetics against 15 *P. vivax* antigens in 34 Thai individuals following symptomatic *P. vivax* infections. 15 *P. vivax* antigens with IgM positivity (> 2 SD above baseline median) at 1-week post-infection were selected and IgM responses over a 9-month period were measured in symptomatic Thai individuals (n=17 timepoints). Horizontal solid lines represent the baseline responses measured in malaria-naïve individuals from 3 different sources (n=274). Data are expressed as the median ± 95% CI.


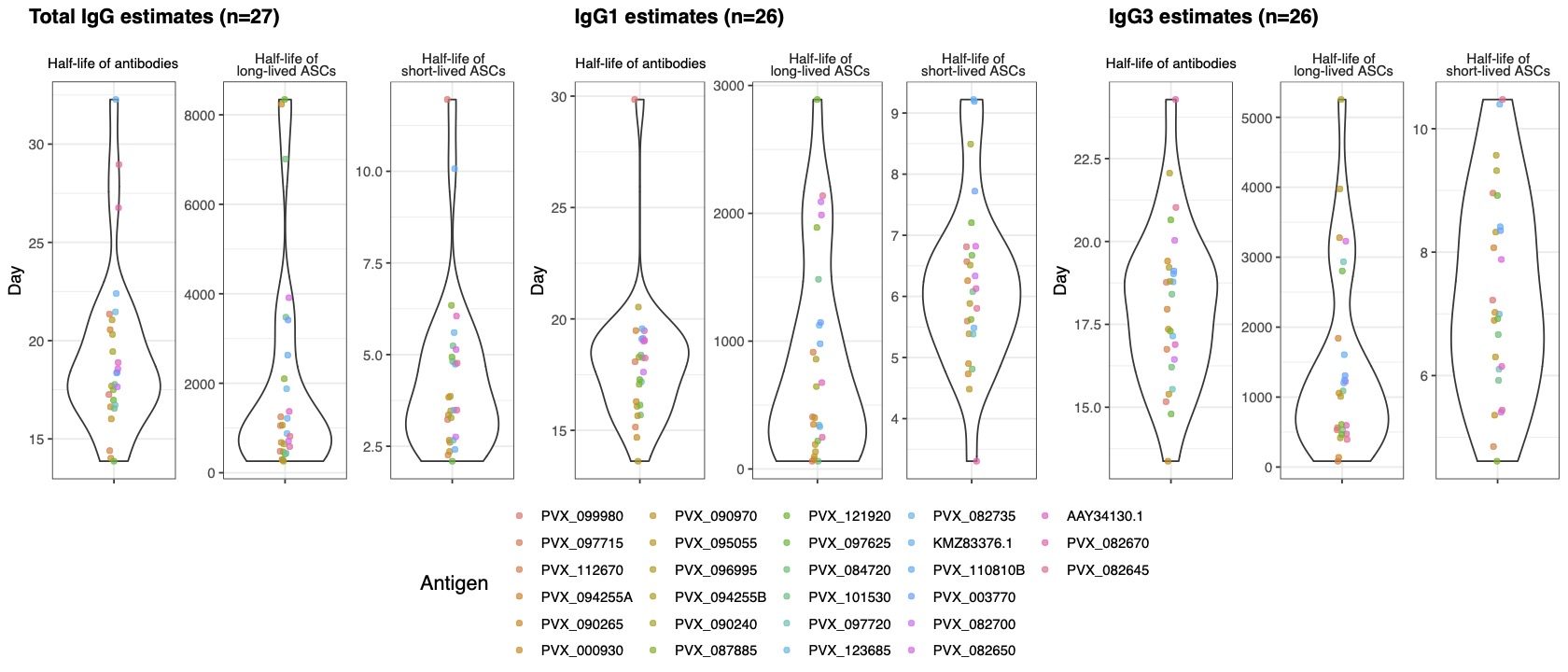


**Figure S7.** Distribution of data for estimated half-lives of antibodies and ASCs against IgG-immunogenic antigens following symptomatic *P. vivax* infections in the Thai symptomatic cohort. The kinetics of total IgG and IgG subclass antibodies against 52 *P. vivax* antigens were determined following symptomatic *P. vivax* infections in Thai individuals in the absence of recurrent infections for 9 months. Mathematical modelling was then used to generate the estimated half-life of antibodies and ASCs for total IgG, IgG1 and IgG3 per antigen. The difference in distribution between antibody isotypes was visualised.


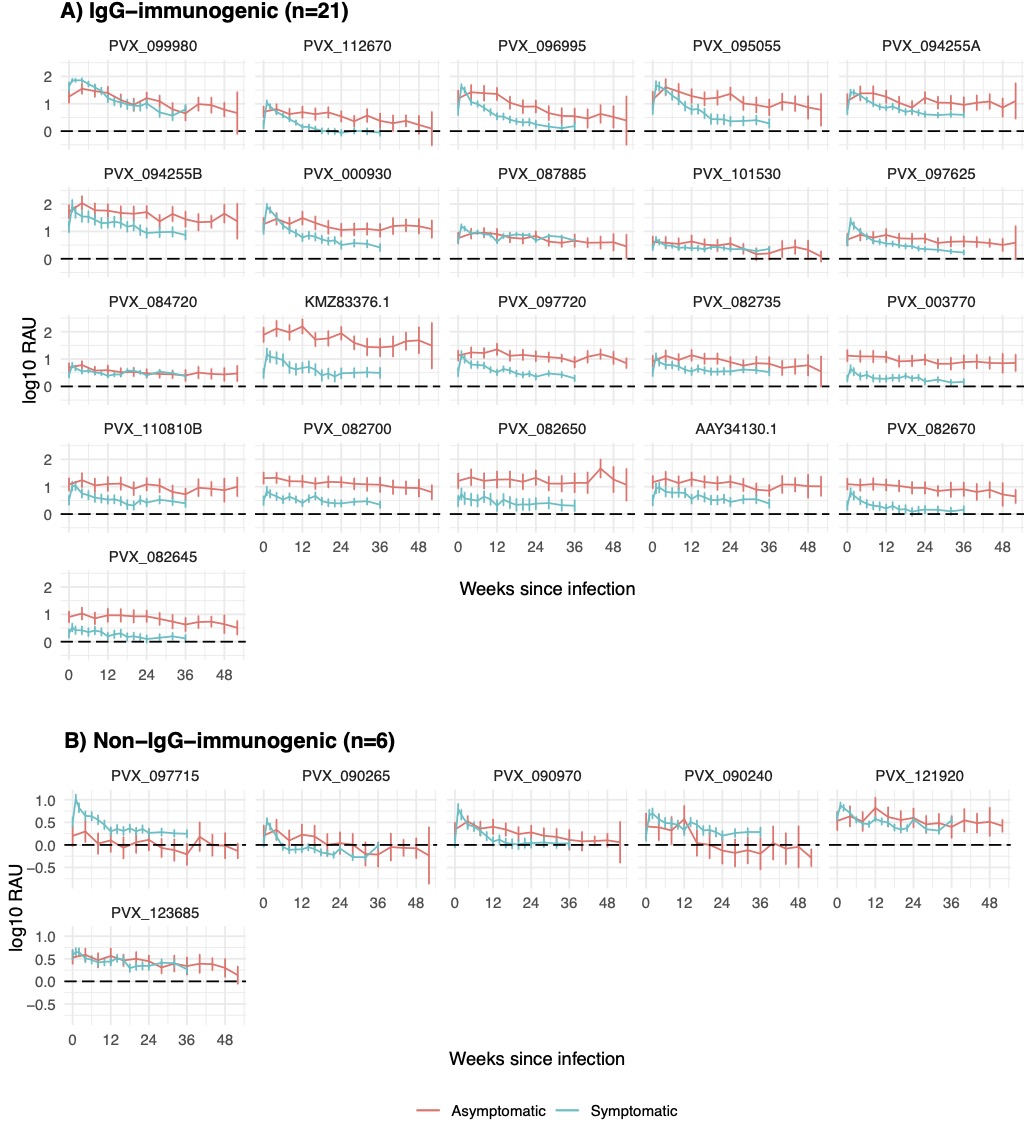


**Figure S8.** Comparison of adjusted total IgG against 27 *P. vivax* antigens following symptomatic or asymptomatic *P. vivax* infections. The kinetics of total IgG antibody against 27 IgG-immunogenic *P. vivax* antigens were determined following both asymptomatic (for a year) and symptomatic (for 9 months) *P. vivax* infections in Thai individuals in the absence of recurrent infections. Antibody data after asymptomatic infection were aligned to the time of infection, and thus sample size diminishes at later time points. Each line and colour represent a study cohort (either asymptomatic or symptomatic) and its specific antibody kinetics following exposure. The observed kinetic profiles were categorised into **A)** IgG-immunogenic (> 1 SD at 1-week post-infection above negative control baseline; n = 21) and **B)** non-IgG-immunogenic (> 1 SD at 1-week post-infection; n = 6) based on the asymptomatic profiles. Data were adjusted by subtracting the median of pooled negative control panels (n=274) and are expressed as median ± 95% CI.
