# Supplementary material for: Naturally acquired antibody kinetics against Plasmodium vivax antigens in people from a low malaria transmission region in western Thailand
Source: BMC Med. 2022 Mar 9;20:89. doi: 10.1186/s12916-022-02281-9 (PMC8904165; doi:10.1186/s12916-022-02281-9)
Supplement: Supplementary file 2 — Additional file 2: Table S1. Immunogenicity of total IgG against 52 P. vivax antigens at 1-week post-infection in Thai symptomatic volunteers. Table S2. P. vivax proteins used in this study. Table S3. Immunogenicity of total IgG against 27 IgG-immunogenic P. vivax antigens at 0-week since infection in Thai asymptomatic volunteers. [file 12916_2022_2281_MOESM2_ESM.docx]

Additional File 2: Tables S1-S3

**Table S1.** Immunogenicity of total IgG against 52 *P. vivax* antigens at 1-week post-infection in Thai symptomatic volunteers.

|  |  |  | | Number of volunteers (%) | | | | |
| --- | --- | --- | --- | --- | --- | --- | --- | --- |
| Protein ID^a^ | Median^b^ | 95% CI^b^ | | 1% of  PNG levels | 5% of  PNG levels | 10% of  PNG levels | 25% of  PNG levels | 50% of  PNG levels |
| PVX_099980 | 20.00 | 9.15 | 20.00 | 34 (1.0) | 34 (1.0) | 34 (1.0) | 34 (1.0) | 17 (0.5) |
| PVX_096995 | 4.99 | 2.40 | 15.29 | 34 (1.0) | 34 (1.0) | 33 (1.0) | 27 (0.8) | 20 (0.6) |
| PVX_088860 | 1.18 | 0.63 | 15.29 | 34 (1.0) | 28 (0.8) | 19 (0.6) | 8 (0.2) | 7 (0.2) |
| PVX_101530 | 1.95 | 0.84 | 3.28 | 34 (1.0) | 32 (0.9) | 23 (0.7) | 15 (0.4) | 8 (0.2) |
| PVX_112680 | 0.43 | 0.26 | 0.97 | 34 (1.0) | 20 (0.6) | 11 (0.3) | 5 (0.1) | 3 (0.1) |
| PVX_097715 | 8.13 | 3.37 | 19.14 | 34 (1.0) | 34 (1.0) | 32 (0.9) | 29 (0.9) | 22 (0.6) |
| PVX_094830 | 0.42 | 0.20 | 1.41 | 34 (1.0) | 18 (0.5) | 13 (0.4) | 8 (0.2) | 2 (0.1) |
| PVX_112675 | 0.36 | 0.25 | 0.52 | 32 (0.9) | 15 (0.4) | 8 (0.2) | 5 (0.1) | 2 (0.1) |
| PVX_112670 | 6.97 | 2.50 | 15.91 | 34 (1.0) | 34 (1.0) | 32 (0.9) | 28 (0.8) | 23 (0.7) |
| PVX_090970 | 5.14 | 1.61 | 13.60 | 34 (1.0) | 32 (0.9) | 29 (0.9) | 24 (0.7) | 18 (0.5) |
| PVX_084720 | 1.94 | 1.04 | 4.07 | 34 (1.0) | 33 (1.0) | 25 (0.7) | 12 (0.4) | 7 (0.2) |
| PVX_003770 | 0.75 | 0.27 | 2.94 | 34 (1.0) | 24 (0.7) | 18 (0.5) | 12 (0.4) | 8 (0.2) |
| PVX_092990 | 2.27 | 1.06 | 5.70 | 34 (1.0) | 33 (1.0) | 29 (0.9) | 22 (0.6) | 11 (0.3) |
| PVX_091710 | 0.76 | 0.59 | 2.29 | 34 (1.0) | 26 (0.8) | 17 (0.5) | 9 (0.3) | 7 (0.2) |
| PVX_087885 | 2.93 | 1.51 | 5.22 | 34 (1.0) | 33 (1.0) | 29 (0.9 | 19 (0.6) | 9 (0.3) |
| PVX_003555 | 0.41 | 0.16 | 1.37 | 34 (1.0) | 20 (0.6) | 15 (0.4) | 9 (0.3) | 6 (0.2) |
| PVX_117385 | 1.65 | 1.08 | 3.88 | 34 (1.0) | 31 (0.9) | 22 (0.6) | 11 (0.3) | 5 (0.1) |
| PVX_090265 | 5.37 | 2.59 | 7.43 | 34 (1.0) | 34 (1.0) | 31 (0.9) | 25 (0.7) | 17 (0.5) |
| PVX_082700 | 0.44 | 0.19 | 2.39 | 27 (0.8) | 15 (0.4) | 12 (0.4) | 10 (0.3) | 7 (0.2) |
| PVX_082650 | 0.43 | 0.12 | 5.06 | 27 (0.8) | 17 (0.5) | 12 (0.4) | 9 (0.3) | 9 (0.3) |
| PVX_094255A^c^ | 5.46 | 2.45 | 7.84 | 34 (1.0) | 34 (1.0) | 29 (0.9) | 29 (0.9) | 21 (0.6) |
| PVX_097680 | 0.49 | 0.16 | 1.39 | 33 (1.0) | 19 (0.6) | 10 (0.3) | 7 (0.2) | 5 (0.1) |
| PVX_001000 | 0.23 | 0.14 | 0.80 | 29 (0.9) | 14 (0.4) | 8 (0.2) | 5 (0.1) | 2 (0.1) |
| PVX_097625 | 2.52 | 0.85 | 5.16 | 34 (1.0) | 30 (0.9) | 28 (0.8) | 19 (0.6) | 12 (0.4) |
| PVX_082670 | 0.33 | 0.14 | 1.69 | 25 (0.7) | 12 (0.4) | 10 (0.3) | 8 (0.2) | 6 (0.2) |
| PVX_099930 | 3.53 | 2.40 | 6.53 | 34 (1.0) | 34 (1.0) | 33 (1.0) | 19 (0.6) | 9 (0.3) |
| PVX_084340 | 1.15 | 0.78 | 4.09 | 34 (1.0) | 27 (0.8) | 17 (0.5) | 10 (0.3) | 7 (0.2) |
| PVX_098915 | 1.75 | 0.89 | 2.24 | 34 (1.0) | 29 (0.9) | 23 (0.7) | 8 (0.2) | 6 (0.2) |
| PVX_088820 | 2.01 | 1.55 | 3.47 | 34 (1.0) | 34 (1.0) | 31 (0.9) | 13 (0.4) | 7 (0.2) |
| PVX_082735 | 1.02 | 0.41 | 2.20 | 32 (0.9) | 21 (0.6) | 16 (0.5) | 10 (0.3) | 7 (0.2) |
| PVX_082645 | 0.25 | 0.15 | 1.69 | 28 (0.8) | 13 (0.4) | 10 (0.3) | 7 (0.2) | 3 (0.1) |
| PVX_117880 | 0.52 | 0.27 | 1.85 | 33 (1.0) | 17 (0.5) | 14 (0.4) | 7 (0.2) | 3 (0.1) |
| PVX_121897 | 3.37 | 2.03 | 7.57 | 34 (1.0) | 34 (1.0) | 30 (0.9) | 20 (0.6) | 13 (0.4) |
| PVX_125728 | 0.45 | 0.25 | 0.71 | 34 (1.0) | 21 (0.6) | 10 (0.3) | 2 (0.1) | 1 (0.0) |
| PVX_090330 | 0.27 | 0.17 | 0.92 | 27 (0.8) | 12 (0.4) | 9 (0.3) | 4 (0.1) | 1 (0.0) |
| PVX_123685 | 1.03 | 0.54 | 2.29 | 34 (1.0) | 24 (0.7) | 13 (0.4) | 9 (0.3) | 3 (0.1) |
| PVX_125738 | 0.65 | 0.41 | 1.83 | 34 (1.0) | 13 (0.4) | 10 (0.3) | 4 (0.1) | 2 (0.1) |
| PVX_097720 | 1.37 | 0.31 | 3.50 | 31 (0.9) | 22 (0.6) | 19 (0.6) | 13 (0.4) | 7 (0.2) |
| PVX_000930 | 5.29 | 1.66 | 11.96 | 34 (1.0) | 34 (1.0) | 32 (0.9) | 25 (0.7) | 20 (0.6) |
| PVX_098585 | 1.62 | 0.78 | 4.39 | 34 (1.0) | 30 (0.9) | 25 (0.7) | 15 (0.4) | 10 (0.3) |
| PVX_098582 | 2.81 | 1.91 | 4.56 | 34 (1.0) | 34 (1.0) | 31 (0.9) | 14 (0.4) | 7 (0.2) |
| PVX_121920 | 2.54 | 1.69 | 3.55 | 34 (1.0) | 34 (1.0) | 30 (0.9) | 15 (0.4) | 4 (0.1) |
| PVX_094255B^c^ | 4.72 | 1.20 | 14.10 | 29 (0.9) | 28 (0.8) | 26 (0.8) | 25 (0.7) | 22 (0.6) |
| PVX_090325 | 0.67 | 0.21 | 2.82 | 34 (1.0) | 19 (0.6) | 15 (0.4) | 9 (0.3) | 6 (0.2) |
| PVX_101590 | 3.47 | 2.09 | 8.45 | 34 (1.0) | 34 (1.0) | 30 (0.9) | 20 (0.6) | 12 (0.4) |
| PVX_110810A^c^ | 0.51 | 0.11 | 1.46 | 29 (0.9) | 21 (0.6) | 15 (0.4) | 9 (0.3) | 6 (0.2) |
| PVX_090240 | 3.70 | 1.10 | 15.50 | 34 (1.0) | 34 (1.0) | 29 (0.9) | 19 (0.6) | 15 (0.4) |
| PVX_088910 | 1.07 | 0.67 | 1.62 | 34 (1.0) | 21 (0.6) | 15 (0.4) | 5 (0.1) | 3 (0.1) |
| PVX_095055 | 5.10 | 1.18 | 17.45 | 34 (1.0) | 30 (0.9) | 28 (0.8) | 21 (0.6) | 18 (0.5) |
| PVX_110810B^c^ | 0.90 | 0.35 | 3.18 | 33 (1.0) | 25 (0.7) | 19 (0.6) | 11 (0.3) | 8 (0.2) |
| AAY34130.1^b^ | 0.37 | 0.15 | 2.49 | 30 (0.9) | 19 (0.6) | 12 (0.4) | 10 (0.3) | 5 (0.1) |
| KMZ83376.1^b^ | 0.97 | 0.11 | 5.04 | 28 (0.8) | 21 (0.6) | 18 (0.5) | 16 (0.5) | 11 (0.3) |

^a^ PlasmoDB codes (http://plasmodb.org/plasmo/), ^b^ Total IgG levels are expressed in relative antibody units (RAU) interpolated from standard curves using a 5PL logistic regression model. Values were multiplied by 1000, ^c^ A and B represent two distinct protein fragments derived from one full-length protein sequence and hence are expressed by identical PlasmoDB code. Abbreviations: 95% CI = 95% confidence interval.

**Table S2.** *P. vivax* proteins used in this study.

| Antigen ID^a^ | Gene Annotation | Expression stage | Protein length (aa) | Construct, aa (size) | Expression System | Purification Method | Amount (μg)^d^ | SP | TMD | GPI | Motif | Predicted Domains | LCR | Reference (PMID) |
| --- | --- | --- | --- | --- | --- | --- | --- | --- | --- | --- | --- | --- | --- | --- |
| PVX_099980 | merozoite surface protein 1 (MSP1) MSP119 | Blood | 1751 | 1622-1729 (108) | WGCF | AC | 0.76 | Yes | Yes | Yes | PNEP | 1 | 12 | 32405064 |
| PVX_096995 | tryptophan-rich antigen (Pv-fam-a) | Blood | 480 | 61-end (420) | WGCF | AC | 0.85 | No | Yes | No | PNEP | 1 | 7 | 32405064 |
| PVX_088860 | sporozoite invasion-associated protein 2 (SIAP2) | Blood, Pre-erythrocytic | 412 | 33-end (380) | WGCF | AC | 0.95 | Yes | Yes | No | PNEP | NP | 5 | 32405064 |
| PVX_101530 | Plasmodium exported protein, unknown function | Blood | 367 | 38-end (330) | WGCF | AC | 0.025 | Yes | Yes | No | PEXEL | NP | 3 | 32405064 |
| PVX_112680 | tryptophan/threonine-rich antigen | Blood | 313 | 33-end (281) | WGCF | AC | 1.2 | Yes | No | No | PNEP | 1 | 1 | 32405064 |
| PVX_097715 | hypothetical protein | Blood | 450 | 20-end (431) | WGCF | AC | 0.35 | Yes | Yes | No | PNEP | NP | 6 | 32405064 |
| PVX_094830 | hypothetical protein, conserved | Blood | 250 | 19-end (232) | WGCF | AC | 0.69 | Yes | No | No | PNEP | NP | 1 | 32405064 |
| PVX_112675 | tryptophan-rich antigen (Pv-fam-a) | Blood | 312 | 33-end (280) | WGCF | AC | 0.47 | Yes | Yes | No | PNEP | 2 | 1 | 32405064 |
| PVX_112670 | tryptophan-rich antigen (Pv-fam-a) | Blood | 335 | 34-end (302) | WGCF | AC | 1.13 | Yes | No | No | PNEP | 1 | 4 | 32405064 |
| PVX_090970 | hypothetical protein, conserved | Unknown | 266 | 20-254 (235) | WGCF | AC | 2 | Yes | Yes | No | PNEP | NP | 3 | 32405064 |
| PVX_084720 | translocon component PTEX150 (PTEX150) | Blood | 908 | 24-908 (885) | WGCF | AC | 0.12 | Yes | No | No | PNEP | NP | 11 | 32405064 |
| PVX_003770 | merozoite surface protein 5 | Blood | 387 | 23-365 (343) | WGCF | AC | 0.025 | Yes | Yes | Yes | PNEP | NP | 2 | 32405064 |
| PVX_092990 | tryptophan-rich antigen (Pv-fam-a) | Blood | 1414 | 1126-1414 (289) | WGCF | AC | 2.6 | Yes | Yes | No | PNEP | 1 | 17 | 32405064 |
| PVX_091710 | hypothetical protein, conserved | Blood | 1689 | 26-884 (859) | WGCF | AC | 0.22 | Yes | No | No | PNEP | 1 | 8 | 32405064 |
| PVX_087885 | rhoptry associated membrane antigen, putative | Blood | 730 | 462-730 (269) | WGCF | AC | 0.15 | Yes | No | No | PNEP | NP | 11 | 32405064 |
| PVX_003555 | Plasmodium exported protein, unknown function | Blood | 1122 | 434-1075 (642) | WGCF | AC | 0.025 | No | Yes | No | PEXEL | 1 | 7 | 32405064 |
| PVX_117385 | phosphatidylinositol-4-phosphate-5-kinase | Blood | 326 | 1-326 (326) | WGCF | AC | 1 | No | No | No | PNEP | NP | 2 | 32405064 |
| PVX_090265 | tryptophan-rich antigen (Pv-fam-a) | Blood | 326 | 1-326 (326) | WGCF | AC | 0.5 | No | Yes | No | PNEP | 1 | 3 | 32405064 |
| PVX_082700 | merozoite surface protein 7 | Blood | 420 | 23-end (397) | WGCF | AC | 0.5 | Yes | No | No | PNEP | 1 | 2 | 32405064 |
| PVX_082650 | merozoite surface protein 7 | Blood | 453 | 24-end (429) | WGCF | AC | 0.35 | Yes | No | No | PNEP | 1 | 4 | 32405064 |
| PVX_094255A^c^ | reticulocyte binding protein 2b (RBP2b) | Blood | 2806 | 1986-2653 (667) | WGCF | AC | 0.7 | Yes | No | No | PNEP | NP | 10 | 32405064 |
| PVX_097680 | merozoite surface protein 3b | Blood | 1016 | 21-end (996) | WGCF | AC | 0.15 | Yes | No | No | PNEP | NP | 17 | 32405064 |
| PVX_001000 | hypothetical protein | Unknown | 668 | 20-end (650) | WGCF | AC | 0.5 | Yes | No | No | PEXEL | NP | 3 | 32405064 |
| PVX_097625 | merozoite surface protein 8 | Blood | 487 | 24-463 (440) | WGCF | AC | 0.175 | Yes | Yes | Yes | PNEP | 2 | 4 | 32405064 |
| PVX_082670 | merozoite surface protein 7 | Blood | 411 | 24-end (388) | WGCF | AC | 0.5 | Yes | No | No | PEXEL | 1 | 5 | 32405064 |
| PVX_099930 | high molecular weight rhoptry protein-2 | Blood | 1369 | 23-387 (365) | WGCF | AC | 0.5 | Yes | No | No | PNEP | NP | 3 | 32405064 |
| PVX_084340 | IMP-specific 5'-nucleotidase | Unknown | 444 | 1-444 (444) | WGCF | AC | 0.65 | No | No | No | PNEP | NP | 1 | 32405064 |
| PVX_098915 | subpellicular microtubule protein 1 (SPM1) | Unknown | 521 | 1-521 (521) | WGCF | AC | 1 | No | No | No | PNEP | NP | 0 | 32405064 |
| PVX_088820 | tryptophan-rich antigen (Pv-fam-a) | Blood | 316 | 58-end (259) | WGCF | AC | 8 | No | Yes | No | PNEP | 1 | 2 | 32405064 |
| PVX_082735 | PvTRAP/SSP2 | Pre-erythrocytic | 556 | 26-493 (468) | WGCF | AC | 0.5 | Yes | Yes | No | PNEP | 1 | 5 | 32405064 |
| PVX_082645 | merozoite surface protein 7 | Blood | 377 | 23-end (355) | WGCF | AC | 0.46 | Yes | No | No | PNEP | 1 | 4 | 32405064 |
| PVX_117880 | rhoptry neck protein 2, putative (RON2) | Blood, Pre-erythrocytic | 2203 | 21-198 (178) | WGCF | AC | 0.75 | Yes | Yes | No | PNEP | NP | 13 | 32405064 |
| PVX_121897 | tryptophan-rich antigen (Pv-fam-a) | Unknown | 275 | 24-end (252) | WGCF | AC | 4 | Yes | No | No | PNEP | 1 | 1 | 32405064 |
| PVX_125728 | tryptophan-rich antigen (Pv-fam-a) | Unknown | 279 | 30-end (250) | WGCF | AC | 16 | Yes | No | No | PNEP | 1 | 2 | 32405064 |
| PVX_090330 | reticulocyte binding protein 2 precursor (PvRBP-2) | Blood | 623 | 31-141 (111) | WGCF | AC | 0.4 | Yes | No | No | PNEP | NP | 4 | 32405064 |
| PVX_123685 | histone-lysine N-methyltransferase | Blood | 1963 | 1320-end (644) | WGCF | AC | 0.65 | No | No | No | PNEP | 3 | 12 | 32405064 |
| PVX_125738 | reticulocyte binding protein 1 precursor | Blood | 786 | 1-786 (786) | WGCF | AC | 0.5 | No | Yes | No | PNEP | NP | 3 | 32405064 |
| PVX_097720 | merozoite surface protein 3a | Blood | 852 | 25-end (828) | WGCF | AC | 0.125 | Yes | Yes | No | PNEP | NP | 14 | 32405064 |
| PVX_000930 | sexual stage antigen s16 | Blood, Sexual | 140 | 31-end (110) | WGCF | AC | 0.25 | Yes | Yes | No | PNEP | NP | 2 | 32405064 |
| PVX_098585 | reticulocyte binding protein 1a (RBP1a) | Blood | 2833 | 160-1170 (1011) | *E. coli* | AC x 2, SEC | 0.6 | Yes | No | No | PNEP | NP | 12 | 26712206 |
| PVX_098582 | reticulocyte binding protein 1b (RBP1b) | Blood | 2608 | 140-1275 (1136) | *E. coli* | AC x 2, SEC | 0.8 | Yes | No | No | PNEP | NP | 14 | 26712206 |
| PVX_121920 | reticulocyte binding protein 2a (RBP2a) | Blood | 2487 | 160-1135 (976) | *E. coli* | AC x 2, SEC | 0.9 | Yes | No | No | PNEP | 1 | 9 | 26712206 |
| PVX_094255B^c^ | reticulocyte binding protein 2b (RBP2b) | Blood | 2806 | 161-1454 (1294) | *E. coli* | AC x 2, SEC | 0.1 | Yes | No | No | PNEP | 1 | 10 | 26712206 |
| PVX_090325 | reticulocyte binding protein 2c (RBP2c non-binding region) | Blood | 2824 | 501-1300 (800) | *E. coli* | AC x 2, SEC | 0.3 | Yes | Yes | No | PNEP | NP | 16 | 26712206 |
| PVX_101590 | reticulocyte-binding protein 2 (RBP2), like (RBP2-P2) | Blood | 641 | 161-641 (481) | *E. coli* | AC x 2, SEC | 1 | Yes | No | No | PNEP | NP | 4 | 28949293 |
| PVX_110810A^c^ | Duffy binding protein (DBP, region 3-5, Sal1 strain) | Blood | 1070 | 193-521 (329) | *E. coli* | AC | 0.2 | Yes | Yes | No | PNEP | 4 | 8 | 19695492 |
| PVX_090240 | cysteine-rich protective antigen, putative (CyRPA) | Blood | 366 | 27-366 (340) | Baculovirus | AC, SEC | 0.5 | Yes | No | No | PNEP | NP | 1 | 26505753 |
| PVX_088910 | GPI-anchored micronemal antigen, putative (GAMA) | Blood | 771 | 22-551 (530) | *E. coli* | AC x 2 | 0.6 | Yes | No | Yes | PNEP | NP | 8 | 19695492 |
| PVX_095055 | Rh5 interacting protein, putative (RIPR) | Blood | 1075 | 552-1075 (524) | *E. coli* | AC x 2, SEC | 0.5 | Yes | No | No | PNEP | 9 | 1 | 24039774 |
| PVX_110810B^c^ | Duffy binding protein (DBP, region 2, Sal1 strain) | Blood | 1070 | 193-521 (329) | *E. coli* | AC, IEC, SEC | 0.36 | Yes | Yes | No | PNEP | 4 | 8 | 19564376; 28949293 |
| AAY34130.1^b^ | Duffy binding protein (DBP, region 2, AH strain) | Blood | 237 | 1-237 (237) | *E. coli* | AC, IEC, SEC | 0.45 | No | No | No | PNEP | 1 | 2 | 19564376 |
| KMZ83376.1^b^ | Erythrocyte binding protein (PvEBPII) | Blood | 786 | 109-432 (324) | *E. coli* | AC, IEC, SEC | 0.1 | No | Yes | No | PNEP | 1 | 3 | 19564376; 28949293 |

^a^PlasmoDB codes (http://plasmodb.org/plasmo/), ^b^GenBank IDs, ^c^A and B represent two distinct protein fragments derived from one full-length protein sequence and hence are expressed by identical PlasmoDB code, ^d^Amount refers to the amount of antigen coupled to non-magnetic microspheres. Abbreviations: aa = Amino acid, WGCF = Wheat germ cell-free, E. coli = Escherichia coli, AC = Affinity chromatography, SEC = Size exclusion chromatography, IEC = Ion exchange chromatography, SP = Signal peptide, TMD = transmembrane domain, GPI = Glycosylphosphatidylinositol anchors, PEXEL = Plasmodium exported element, PNEP = PEXEL-negative exported protein, NP = Non-predicted, LCR = Low complexity region.

**Table S3.** Immunogenicity of total IgG against 27 IgG-immunogenic *P. vivax* antigens at 0-week since infection in Thai asymptomatic volunteers.

| Number of volunteers (%) | | | | | | | | |
| --- | --- | --- | --- | --- | --- | --- | --- | --- |
| Protein ID^a^ | Median^b^ | 95% CI^b^ | | 1% of  PNG levels | 5% of  PNG levels | 10% of  PNG levels | 25% of  PNG levels | 50% of  PNG levels |
| PVX_099980 | 5.05 | 1.29 | 13.59 | 30 (1.0) | 28 (0.9) | 25 (0.8) | 21 (0.7) | 16 (0.5) |
| PVX_096995 | 1.88 | 0.27 | 9.17 | 27 (0.9) | 22 (0.7) | 19 (0.6) | 15 (0.5) | 11 (0.4) |
| PVX_101530 | 1.52 | 0.67 | 3.40 | 30 (1.0) | 27 (0.9) | 20 (0.7) | 13 (0.4) | 8 (0.3) |
| PVX_097715 | 1.27 | 0.32 | 3.18 | 29 (1.0) | 17 (0.6) | 10 (0.3) | 7 (0.2) | 3 (0.1) |
| PVX_112670 | 3.24 | 1.28 | 14.14 | 29 (1.0) | 28 (0.9) | 20 (0.7) | 15 (0.5) | 10 (0.3) |
| PVX_090970 | 1.93 | 1.47 | 4.40 | 30 (1.0) | 28 (0.9) | 24 (0.8) | 16 (0.5) | 6 (0.2) |
| PVX_084720 | 1.50 | 0.72 | 3.04 | 30 (1.0) | 29 (1.0) | 22 (0.7) | 16 (0.5) | 8 (0.3) |
| PVX_003770 | 2.18 | 0.70 | 4.10 | 29 (1.0) | 24 (0.8) | 22 (0.7) | 13 (0.4) | 6 (0.2) |
| PVX_087885 | 1.05 | 0.68 | 4.21 | 30 (1.0) | 30 (1.0) | 29 (1.0) | 28 (0.9) | 19 (0.6) |
| PVX_090265 | 2.78 | 0.84 | 7.26 | 30 (1.0) | 30 (1.0) | 29 (1.0) | 27 (0.9) | 19 (0.6) |
| PVX_082700 | 1.25 | 0.63 | 2.79 | 29 (1.0) | 27 (0.9) | 22 (0.7) | 14 (0.5) | 13 (0.4) |
| PVX_082650 | 1.12 | 0.33 | 12.07 | 26 (0.9) | 16 (0.5) | 18 (0.6) | 10 (0.3) | 6 (0.2) |
| PVX_094255A^c^ | 2.63 | 0.58 | 7.58 | 30 (1.0) | 30 (1.0) | 28 (0.9) | 27 (0.9) | 25 (0.8) |
| PVX_097625 | 0.51 | 0.39 | 1.67 | 26 (0.9) | 11 (0.4) | 9 (0.3) | 6 (0.2) | 4 (0.1) |
| PVX_082670 | 0.70 | 0.36 | 1.98 | 30 (1.0) | 26 (0.9) | 21 (0.7) | 12 (0.4) | 7 (0.2) |
| PVX_082735 | 0.80 | 0.60 | 3.52 | 30 (1.0) | 17 (0.6) | 13 (0.4) | 8 (0.3) | 5 (0.2) |
| PVX_082645 | 0.63 | 0.38 | 2.19 | 29 (1.0) | 13 (0.4) | 9 (0.3) | 4 (0.1) | 3 (0.1) |
| PVX_123685 | 0.80 | 0.48 | 1.18 | 30 (1.0) | 26 (0.9) | 22 (0.7) | 11 (0.4) | 4 (0.1) |
| PVX_097720 | 1.22 | 0.63 | 2.97 | 24 (0.8) | 4 (0.1) | 4 (0.1) | 2 (0.1) | 0 (0.0) |
| PVX_000930 | 1.07 | 0.57 | 4.40 | 20 (0.7) | 8 (0.3) | 4 (0.1) | 1 (0.0) | 0 (0.0) |
| PVX_090240 | 2.37 | 0.38 | 5.90 | 30 (1.0) | 26 (0.9) | 19 (0.6) | 15 (0.5) | 4 (0.1) |
| PVX_110810B^c^ | 0.93 | 0.36 | 3.30 | 29 (1.0) | 18 (0.6) | 10 (0.3) | 5 (0.2) | 1 (0.0) |
| AAY34130.1 | 0.56 | 0.28 | 1.28 | 23 (0.8) | 7 (0.2) | 5 (0.2) | 1 (0.0) | 1 (0.0) |
| KMZ83376.1 | 5.56 | 1.05 | 12.12 | 27 (0.9) | 21 (0.7) | 16 (0.5) | 7 (0.2) | 2 (0.1) |
| PVX_095055 | 1.48 | 0.27 | 9.20 | 30 (1.0) | 24 (0.8) | 22 (0.7) | 18 (0.6) | 16 (0.5) |
| PVX_121920 | 1.16 | 0.68 | 2.34 | 30 (1.0) | 30 (1.0) | 28 (0.9) | 20 (0.7) | 8 (0.3) |
| PVX_094255B^c^ | 2.64 | 0.94 | 5.82 | 26 (0.9) | 20 (0.7) | 13 (0.4) | 5 (0.2) | 1 (0.0) |

^a^ PlasmoDB codes (http://plasmodb.org/plasmo/), ^b^ Total IgG levels are expressed in relative antibody units (RAU) interpolated from standard curves using a 5PL logistic regression model. Values were multiplied by 1000, ^c^ A and B represent two distinct protein fragments derived from one full-length protein sequence and hence are expressed by identical PlasmoDB code. Abbreviations: 95% CI = 95% confidence interval
